# Supplementary material for: Content-rich biological network constructed by mining PubMed abstracts
Source: BMC Bioinformatics. 2004 Oct 8;5:147. doi: 10.1186/1471-2105-5-147 (PMC528731; doi:10.1186/1471-2105-5-147)
Supplement: Additional File 2 — The original results of the above study (non-essential files are deleted to keep the file size under the limit set by BMC bioinformatics). [file 1471-2105-5-147-S2.bz2 › chilibotAdditionalFile2/dip10/497ID10187774E10660/html/TGFB1_ACVRL1.html]

 


 **TGFB1** and **ACVRL1** 
  
Found 99 abstracts in PubMed, retrieved 10.  
 

 What does Google say? 
 PDF only 
| .edu only 

---

**Interactive relationship** (e.g. stimulation, inhibition, etc)

**Neutral relationship**- ALK  [ **ACVRL1** ]  5 and ALK 1  [ **ACVRL1** ]  mediate TGF beta  [ **TGFB1** ]  signals through distinct Smad proteins, i.e., Smad2 Smad3 and Smad1 Smad5, respectively.  Ref: 12384983 J Cell Physiol, 2002
- Activin receptor like kinase 5 ALK  [ **ACVRL1** ]  5 is a TGF beta  [ **TGFB1** ]  type I receptor expressed in various TGF beta  [ **TGFB1** ]  responsive cells.  Ref: 12384983 J Cell Physiol, 2002
- In endothelial cells, TGF beta  [ **TGFB1** ]  binds to two distinct type I receptor serine threonine kinases, ALK  [ **ACVRL1** ]  5 and ALK 1  [ **ACVRL1** ] .  Ref: 12297674 Sci STKE, 2002
- Recent studies have identified mutations of the bone morphogenetic protein receptor 2 BMPR2 gene and the activin receptor like kinase 1  [ **ACVRL1** ]  ALK1  [ **ACVRL1** ]  gene, that affect the transforming growth factor beta TGF beta  [ **TGFB1** ]  receptor superfamily, a group of transmembrane signaling molecules with serine threonine kinase activity that are involved in the regulation of cell growth.  Ref: 12624796 Z Rheumatol, 2003
- Activin receptor like kinase 1  [ **ACVRL1** ]  ALK 1  [ **ACVRL1** ] , the gene mutated in HHT  [ **ACVRL1** ]  type 2 HHT2  [ **ACVRL1** ] , is a serine threonine kinase receptor type I of the TGF beta  [ **TGFB1** ]  superfamily, specifically expressed on endothelial cells.  Ref: 12700602 Eur J Hum Genet, 2003
- In contrast, ALK 1  [ **ACVRL1** ]  functions as a TGF beta  [ **TGFB1** ]  type I receptor in endothelial cells, and is responsible for human hereditary hemorrhagic telangiectasia HHT  [ **ACVRL1** ]  type II.  Ref: 12384983 J Cell Physiol, 2002
- LXRbeta subsequently modulates signaling by ALK 1  [ **ACVRL1** ]  and the closely related TGF beta  [ **TGFB1** ]  receptor, ALK  [ **ACVRL1** ]  2, as demonstrated by specific and potent inhibition of ALK 1  [ **ACVRL1** ]  and ALK  [ **ACVRL1** ]  2 mediated transcriptional responses, establishing LXRbeta as a potential modulator mediator of ALK 1  [ **ACVRL1** ]  ALK  [ **ACVRL1** ]  2 signaling.  Ref: 12393874 J Biol Chem, 2002
- Immunohistochemistry demonstrated that, compared to controls, shunt lambs have increased TGF beta1  [ **TGFB1** ]  and TbetaRI expression in the smooth muscle layer, and increased TGF beta1  [ **TGFB1** ]  and ALK 1  [ **ACVRL1** ]  in the endothelium of small pulmonary arteries < ; 200 um at 1 and 4 weeks of age.  Ref: 12665463 Am J Physiol Lung Cell Mol Physiol< MedlineTA>Am J Physiol Lung Cell Mol Physiol, 2002

**Non-interactive relationship** (e.g. studied together, co-existance, homology, etc.)

- The transforming growth factor beta TGF beta  [ **TGFB1** ]  receptor, ALK 1  [ **ACVRL1** ] , is expressed specifically on endothelial cells and is essential for angiogenesis, as demonstrated by its targeted deletion in mice and its mutation in the human disease hereditary hemorrhagic telangiectasia.  Ref: 12393874 J Biol Chem, 2002
- TGF beta1  [ **TGFB1** ] , ALK 1  [ **ACVRL1** ] , ALK  [ **ACVRL1** ]  5, and TbetaRII were also detected by immunohistochemistry.  Ref: 12883979 Pediatr NephrolPediatr Nephrol, 2002
- These results revealed some new targets of TGF beta  [ **TGFB1** ]  in endothelial cells, and differences in transcriptional regulation patterns between ALK 1  [ **ACVRL1** ]  and ALK  [ **ACVRL1** ]  5.  Ref: 12384983 J Cell Physiol, 2002
- To determine the potential role of TGF beta1  [ **TGFB1** ]  in this vascular remodeling, we then investigated vascular changes in expression and localization of TGF beta1  [ **TGFB1** ]  and its receptors TbetaRI, ALK 1  [ **ACVRL1** ] , and TbetaRII in the lungs of shunted and age matched control lambs at 1 day,, and 8 weeks of life.  Ref: 12665463 Am J Physiol Lung Cell Mol Physiol< MedlineTA>Am J Physiol Lung Cell Mol Physiol, 2002
- Enalapril decreased renal TGF beta1  [ **TGFB1** ] , ALK 1  [ **ACVRL1** ] , and ALK  [ **ACVRL1** ]  5 protein expression P< ; 0.05.  Ref: 12883979 Pediatr NephrolPediatr Nephrol, 2002
- Although ALK 1  [ **ACVRL1** ]  and another endothelial specific TGF beta  [ **TGFB1** ]  receptor, endoglin, both bind TGF beta  [ **TGFB1** ]  with identical isoform specificity and form a complex together, neither has been shown to signal in response to TGF beta  [ **TGFB1** ] , and the mechanism by which these receptors signal in endothelial cells remains unknown.  Ref: 12393874 J Biol Chem, 2002
- This study was designed to investigate the relationship between renal growth impairment and the expression of transforming growth factor beta1  [ **TGFB1** ]  TGF beta1  [ **TGFB1** ] , TGF beta  [ **TGFB1** ]  receptor I TbetaRI, activin like kinase ALK 1  [ **ACVRL1** ]  and ALK  [ **ACVRL1** ]  5, and TGF beta  [ **TGFB1** ]  receptor II TbetaRII.  Ref: 12883979 Pediatr NephrolPediatr Nephrol, 2002
- Newborn rat pups were treated with enalapril 30 mg kg per day or vehicle for 7 days, and kidneys were removed for Western blotting of TGF beta1  [ **TGFB1** ] , ALK 1  [ **ACVRL1** ] , ALK  [ **ACVRL1** ]  5, and TbetaRII, and for RT PCR of ALK  [ **ACVRL1** ]  5 and TbetaRII.  Ref: 12883979 Pediatr NephrolPediatr Nephrol, 2002
- These results indicate that ACE inhibition in the developing kidney decreases TGF beta1  [ **TGFB1** ] , ALK 1  [ **ACVRL1** ] , and ALK  [ **ACVRL1** ]  5 expression, which may account for renal growth impairment.  Ref: 12883979 Pediatr NephrolPediatr Nephrol, 2002
- Western blots demonstrated that TGF beta1  [ **TGFB1** ]  and ALK 1  [ **ACVRL1** ]  expression were elevated in the shunt as compared to control lambs at 1 and 4 weeks of age P< ; 0.05.  Ref: 12665463 Am J Physiol Lung Cell Mol Physiol< MedlineTA>Am J Physiol Lung Cell Mol Physiol, 2002
